# BCL2: a 30-year tale of life, death and much more to come

**DOI:** 10.1038/cdd.2017.189

**Published:** 2017-11-10

**Authors:** Francesca Pentimalli

**Affiliations:** 1 Centro Ricerche Oncologiche Mercogliano (CROM), Istituto Nazionale Tumori – IRCCS – Fondazione G. Pascale, Naples, Italy

The past three decades of research on *BCL2*, the founding member of a family of apoptotic proteins and archetype of cell death inhibitors, have been tremendously insightful, culminating in the recent successful clinical translation of new therapeutic approaches exploiting cell death pathways (Figure 1). However, to maximize the chances of providing long-term therapeutic benefit to patients with cancer and other diseases, there is still an urgent need to gain a thorough understanding of the mechanisms underlying cell death. This issue of *Cell Death & Differentiation* brings together a set of reviews focused on the crucial role of BCL2 and its protein family in the regulation of apoptosis, with emphasis on unresolved issues, likely to have a profound impact on our understanding of this biological process.

*BCL2* was originally identified in Croce’s lab in 1984 as the target within the breakpoint region of the t(14;18) translocation carried by patients with the follicular variant of B-cell lymphoma, from which it takes its name.^1^ Such chromosomal rearrangement places the immunoglobulin heavy-chain enhancers near the *BCL2* promoter resulting in *BCL2* overexpression. The discovery of *BCL2*, and its function as inhibitor of apoptosis by Vaux,^2^ laid the foundation for the identification of cell death mechanisms across different organisms. It also heralded the key discovery of a new cancer mechanism: some genes contribute to cancer development by inducing cell death escape, rather than cell proliferation, which afterward was recognized as one of the cancer hallmarks.^3^ Here Croce and colleagues recall those early steps and show how the same powerful approach of positional cloning led, years later, to the identification of the first microRNAs known to underlie cancer development.^4^ They were investigating the 13q chromosomal deletion frequently found in patients with chronic lymphocytic leukemia (CLL), the most common human leukemia, in the search for an elusive candidate cancer gene. The analysis of a few CLL specimens allowed them to narrow down the deletion to a 30 kb region which, disappointingly, did not contain any protein-coding gene. This region however contained a cluster of two microRNAs, miR-15a and miR-16, on which they zeroed in. When they found *BCL2* listed as a top predicted target of miR-15/-16, it turned as an immediate pick among proteins involved in B-cell transformation. Indeed they went on demonstrating that the BCL2 overexpression observed in over 70% of CLLs was mainly caused by the loss of miR-15/-16, occurring through deletion or downregulation. Here the authors hail the encouraging results on the efficacy of the BCL2 targeting drug ABT-199 (venetoclax) approved by the FDA in 2016 for the treatment of refractory CLL, and raise important questions as to whether other crucial targets of miR-15/-16 have a role in CLL and could be tackled in combination with BCL2-targeting drugs and whether regulation of miR-15/-16 itself might be translated in the future into a potential pharmaceutical approach against CLL.^4^

The *BCL2* protein family has grown larger over the last years counting 30 members each containing from one to four BCL2 homology (BH) domains and categorized into three functional classes: the pro-survival members (including BCL2 and its closest relatives BCLXL, MCL1 and others) recognized as ‘guardians’ the pro-apoptotic ‘effectors’ (including BAX, BAK and BOK); and the pro-apoptotic ‘BH3-only’ proteins acting as ‘initiators’ (including BIM, BID, PUMA, NOXA and others). BCL2 family proteins control the mitochondrial apoptotic pathway, which is engaged both by physiological stimuli during development or by a myriad of cytotoxic cues. The interaction between BCL2 pro- and anti-apoptotic family members sets the apoptotic threshold acting as a tripartite switch that determines the life/death decision:^5, 6^ once the balance is tipped toward the formation of BAX and BAK oligomers, the cell is committed to suicide. BAX and BAK assembly in fact leads to the formation of pores within the mitochondrial outer membrane causing its permeabilization (MOMP) and providing a getaway for apoptogenic molecules, whose release from the mitochondrial intermembrane space into the cytosol, triggers, in turn, a caspase cascade ultimately leading to cell death.

Mechanistic insights from structural studies and sequence homology have allowed to draw a clear picture of the interactions between BCL2 family members, although there are still many open questions. The multi-BH domain family members (including both pro-survival proteins and the pre-activated forms of BAX and BAK) adopt a globular structure with a characteristic hydrophobic surface groove that mediates the interaction with the BH3 domain of the pro-apoptotic family members. Owing to these intrinsic properties, in healthy cells, pro-survival members keep their pro-apoptotic relatives in check. Following apoptotic cues however, the upregulation of BH3-only proteins favors their binding to pro-survival members, which sets free the pore formers BAX and BAK. Some BH3-only proteins can also directly activate the pore formers, inducing MOMP.^6, 7, 8^ On the basis of this functional dichotomy, BH3-only proteins have been further classified into ‘sensitizers’, which lead to MOMP indirectly (such as BAD and NOXA), and ‘activators’, which directly trigger BAX and BAK oligomerization (such as BID and BIM). The boundaries between these two classes however are blurred, with some activator BH3-only proteins reportedly capable of binding both pore formers and anti-apoptotic proteins, giving rise to different models of apoptotic switch regulation by the BCL2 family, as discussed in.^7, 8, 9^ Moreover, the BH3 domain is not a simple passe-partout for all the BCL2 family member interactions: other residues or motifs might allow specific interactions, stressing the importance of the use of full-length proteins rather than peptides limited to the BH3 region, and also of physiological protein concentrations, to determine relevant interactions, as argued by Andrews and colleagues in this issue.^7^ Here the authors highlight how BCL2 protein interactions depend on protein concentration and on the affinity between members that are both largely affected by protein interaction with intracellular membranes, which are often neglected experimentally.^7^ Adding further complexity, the cellular context, the nature of apoptotic cues (in terms of stress type and intensity) and posttranslational modifications all contribute to dictating the interactions among the three subgroups of the BCL2 family.^7^

The mechanisms acting upstream, during and after MOMP are also being intensively investigated as each step could offer therapeutic opportunities to turn the apoptotic cell machinery into a precision medicine strategy. In particular, targeted release of BAX and BAK ability to perforate the MOM might be harnessed as a powerful killing approach. However, the exact mechanisms underlying pore formation are still a matter of debate.^6, 7, 8^ The precise sequence of events and interactions leading to the formation of BAX/BAK homo-oligomers at the MOM, their transient interaction with BH3-only proteins and the nature, whether lipidic or proteinaceous, of the pores themselves, still need to be elucidated as discussed herein.^6, 7, 8^ Also, Kalkavan and Green examine the role of BOK, which seems to function as an additional, non-canonical MOMP effector, acting independently of BAX and BAK in certain contexts.^8^

But what happens next, after the MOM is pierced? Is the release of molecules (such as citochrome c and SMAC/DIABLO), which reside within the inner mitochondrial space, coordinated? Or are they released independently? Which factors regulate their release is equally a matter of current investigation and unravelling these mechanisms could help to identify patients who could benefit most from the use of SMAC mimetics.^8^

Another challenge brought up here by Green is to understand which features enable the cell to complete its deadly task. Once thought to be an ‘all or nothing’ phenomenon, the output of MOMP seems to be more nuanced: most but not all mitochondria within a cell can undergo MOMP (incomplete MOMP) or just a few mitochondria can be permeated (minority MOMP) when exposed to sublethal cellular stress. In these cases, the outcome of the cell seems likely dependent on the amount of caspase activation but cell survival could come at the high cost of bearing damaged DNA. In the long run, while from an evolutionary perspective such stress-induced genetic diversity could be advantageous, it could also promote tumorigenesis or cancer resistance.^8^

Interestingly, as traced back by Strasser and Vaux in this issue, the origin of the BCL2 family might have occurred with the appearance of the apoptotic effectors in unicellular organisms. Apoptosis in unicellular organisms could have likely provided an advantage serving as an altruistic defense against pathogens. According to this account, subsequent gene duplication and mutations might have given rise to the other family members in multicellular organisms endowing them with a deadly tool to shape development, regulate cell number homeostasis and respond to stress.^10^ While the successful induction of apoptosis is crucial to eliminate undesired cells, it is also critical to protect long-lived cell types and progenitor cells during development. Indeed, we now know that several pro-survival BCL2 family members have developmental roles in a variety of tissue types. Indeed, Opferman and Khotari, in this issue, describe how gene-targeting approaches in animal models have proved to be instrumental to our understanding of how BCL2-mediated mechanisms preserve specific cell lineages at different stages during development.^11^ Our basic understanding of such developmental functions will be important to establish whether the BCL2 family of proteins can be targeted therapeutically (for example, they are currently being investigated as therapeutic targets for neurological conditions)^11^ and to anticipate potential toxicities (as epitomized by BCL-XL, which promotes the survival of mature platelets and, consistently, its targeting by navitoclax was responsible for the dose-limiting BAX- and BAK-mediated thrombocytopenia observed in cancer patients).^6^

As mentioned above, the wealth of information on the BCL2 family structure and function provided a fertile ground for the development of small molecules BH3 mimetics, which mimic the binding of BH3 peptides to the hydrophobic groove of anti-apoptotic proteins thereby displacing both BH3-only proteins and active BAX/BAK from the constraint of pro-survival members. Somewhat surprisingly, considering that cancer cells are usually resistant to apoptosis, many tumors are more prone to die than their normal counterparts owing to the high expression of pro-apoptotic proteins induced by multiple stresses upon transformation. To survive, cancer cells become addicted to the expression of anti-apoptotic BCL2 proteins but such addiction turns into their Achille’s heel. Indeed, inhibition of BCL2 through BH3 mimetics opened a new era in cancer therapy providing the anticancer arsenal with a new class of agents with enormous potential and considerable advantages as discussed by Adams and Cory.^6^ Although various hurdles might hamper their application, especially for solid tumors, the road ahead has begun to be paved: Montero and Letai^9^ show how BH3 profiling, by dissecting the cell apoptotic assets, can reliably identify tumor dependency on specific pro-survival proteins guiding precisely the therapeutic use of BH3 mimetics, many of which are in the clinical development pipeline.^12^ Overall, it seems that the best has yet to come for turning death into better lives.

## Figures and Tables

**Figure 1:**
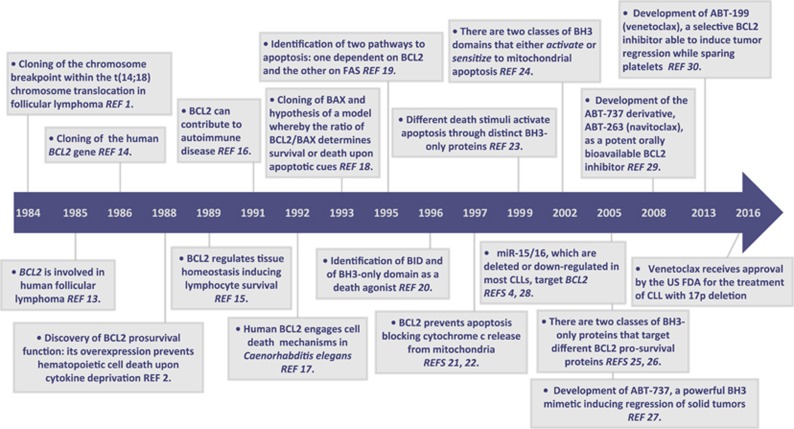
Timeline of breakthrough discoveries from BCL2 identification to BCL2-based clinical therapeutics. Key milestones are indicated amongst all the discoveries achieved across this long road to the development of BCL2-based therapeutics. Many other landmark studies, such as those reporting the identification of other family members, or studies in other species that helped to characterize the mechanisms of mitochondrial apoptosis, as well as many others, are not reported owing to space constraints. FDA: Food and Drug Administration.
